# Solid Phase Extraction Methodology for Robust Isotope Analysis of Atmospheric Ammonium

**DOI:** 10.1021/acsearthspacechem.3c00375

**Published:** 2024-04-15

**Authors:** Alexandra B. MacFarland, Wendell W. Walters, Meredith G. Hastings

**Affiliations:** † Department of Earth, Environmental, and Planetary Sciences Brown University 324 Brook Street, Box 1846 Providence, Rhode Island 02912, United States; ‡ Institute at Brown for Environment and Society Brown University 85 Waterman St Providence, Rhode Island 02912, United States; § Department of Chemistry and Biochemistry University of South Carolina 631 Sumter Street Columbia, South Carolina 29208, United States

**Keywords:** ammonium, atmosphere, purification, stable isotope, nitrogen deposition

## Abstract

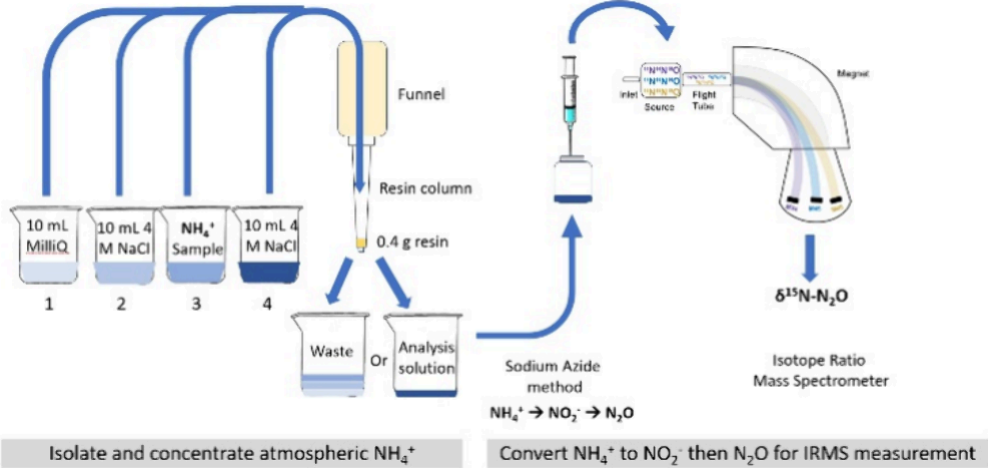

The stable nitrogen isotope composition (δ^15^N) of atmospheric ammonia (NH_3_) and ammonium (NH_4_^+^) has emerged as a potent tool for improving our understanding of the atmospheric burden of reduced nitrogen. However, current chemical oxidation methodologies commonly utilized for characterizing δ^15^N values of NH_4_^+^ samples have been found to lead to low precision for low concentration (i.e., < 5 μmol L^–1^) samples and often suffer from matrix interferences. Here, we present an analytical methodology to extract and concentrate NH_4_^+^ from samples through use of a sample pretreatment step using a solid phase extraction technique involving cation exchange resins. Laboratory control tests indicated that 0.4 g of cation exchange resin (Biorad AG-50W) and 10 mL of 4 M sodium chloride extraction solution enabled the complete capture and removal of NH_4_^+^. Using this sample pretreatment methodology, we obtained accurate and precise δ^15^N values for NH_4_^+^ reference materials and an in-house quality control sample at concentrations as low as 1.0 μM. Additionally, the sample pretreatment methodology was evaluated using atmospheric aerosol samples previously measured for δ^15^N-NH_4_^+^ (from Changdao Island, China), which indicated an excellent δ^15^N-NH_4_^+^ match between sample pretreatment and no treatment (*y* = (0.98 ± 0.05)*x* + (0.11 ± 0.6), *R*^2^ = 0.99). Further, this methodology successfully extracted NH_4_^+^ from aerosol samples and separated it from present matrix effects (samples collected from Oahu, Hawaii; pooled standard deviation δ^15^N-NH_4_^+^ = ± 0.5‰,*n* = 16 paired samples) that without pretreatment originally failed to quantitatively oxidize to nitrite for subsequent δ^15^N isotope analysis. Thus, we recommend applying this sample pretreatment step for all environmental NH_4_^+^ samples to ensure accurate and precise δ^15^N measurement.

## Introduction

Reduced inorganic nitrogen (e.g., NH_*x*_ (ammonia (NH_3_) + ammonium (NH_4_^+^))) is a readily bioavailable form of nitrogen (N), with its rate of loading into the atmosphere increasing over time.^1^ NH_*x*_ can be difficult to track in the environment due to its short lifetime (hours to days)^2^ and its multitude of emission sources and fates (dry deposition, cloud scavenging, formation of NH_4_^+^ aerosols, bidirectional exchange). However, distinguishing between NH_3_ sources from atmospheric deposition is essential to understanding the effects of human activities and natural emissions on various environments.^1^

The stable nitrogen isotope composition of NH_4_^+^ has been shown to be a useful tool for categorizing and detailing the sources and chemistry of reactive N in the environment.^3−7^ This is because different sources of NH_4_^+^ tend to have unique nitrogen isotope compositions.^1,4^ The stable nitrogen isotope composition is typically reported using delta notation (δ^15^N) and is determined as follows:
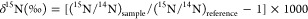
1where the (^15^N/^14^N)_reference_ is air-N_2_. Physical, chemical, and biological processes discern between the two isotopes (^15^N and ^14^N), which result in small but measurable differences in the ratio of ^15^N:^14^N among the inorganic forms of N (e.g., NH) observed in the environment emitted by various sources.^8^ For example, continental and marine aerosol NH_4_^+^ have different observed δ^15^N values, making the tracking of anthropogenic and natural emissions of NH_3_ possible (e.g., marine sources are typically lower in δ^15^N compared to continental sources).^1,3,9,10^ Previous work has indicated that aerosols collected from the remote Atlantic Ocean had low δ^15^N-NH_4_^+^ values that ranged from −8 to −5‰ associated with marine activity and high δ^15^N-NH_4_^+^ up to 10‰ associated with the transport of air masses deriving from continental regions.^3^

Due to the low concentrations of NH_4_^+^ in many environments, it has been historically difficult to measure the δ^15^N of environmental NH_4_^+^ samples. A significant development in our ability to utilize δ^15^N of NH_4_^+^ was the methodology advancement of quantitatively oxidizing NH_4_^+^ to nitrite (NO_2_^–^) using chemical methods, coupled to an established protocol for the conversion of NO_2_^–^ to nitrous oxide gas (N_2_O) for routine δ^15^N analysis for as little as 10 nmol of NH_4_^+^.^11^ This method had been suggested to be a robust technique to quantify δ^15^N of NH_4_^+^ for sample concentrations as low as 0.5 μM. However, recent work has reported a significant reagent blank associated with the chemical method used to oxidize NH_4_^+^ to NO_2_^–^; thus, recommending that this method can only be applied for NH_4_^+^ concentrations above 2 μmol L^–1^, limiting our ability to accurately measure δ^15^N for diluted NH_4_^+^ concentrations.^5,9^

Further, the chemical method for δ^15^N-NH_4_^+^ determination was developed for and tested on freshwater and saltwater samples and was found to have no significant matrix interferences during the conversion of NH_4_^+^ to NO_2_^–^ to N_2_O^11^. Since this method’s development, it has been applied to a range of NH_4_^+^ samples including precipitation, aerosols, and denuded gas.^7,12,13^ Many studies assume successful NH_4_^+^ oxidation to NO_2_^–^ without direct confirmation, but when these checks are performed, quantitative oxidation is often not found. Further, incomplete oxidation of NH_4_^+^ to NO_2_^–^ can induce isotope fractionation resulting in a δ^15^N bias. For example, it was recently shown that NH_4_^+^ extracted from NH_3_ collected on an acid-coated denuder failed to quantitatively oxidize for 7.2% of samples.^6^ More alarmingly, aerosol NH_4_^+^ extracted from aerosol filters failed to oxidize for 38% of samples.^6^ This oxidation failure is likely not caused because of the media and/or acid matrices used for NH_3_ or NH_4_^+^ collection as previously works have indicated no interference when collecting pure NH_3_ or particulate NH_4_^+^ from laboratory investigations,^12,13^ but instead likely reflect the collection of other interferences from the environment complicating the analysis of δ^15^N. Recently in our lab, NH_4_^+^ extracted from aerosol samples (*n* = 57) from Oahu, Hawaii, and NH_4_^+^ extracted from throughfall and soils all failed to oxidize to NO_2_^–^, motivating our search for a sample pretreatment step that can be utilized for the concentration and purification of NH_4_^+^ for high throughput δ^15^N analysis.

Sample pretreatment may therefore be necessary for accurate and robust measurements of δ^15^N-NH_4_^+^. One common approach has been using the microdiffusion method, which attempts to quantitatively isolate NH_3_ from solution via volatilization from solution and collection on an acid-coated filter.^14^ While this could be a suitable approach for concentration of NH_4_^+^ in atmospheric samples, it is time-consuming (requires weeks to complete) and labor intensive and can require significant sample volumes. Additionally, this method requires extensive filter wrapping/taping which can introduce a higher likelihood of human error.^14,15^ Recently, a solid phase extraction technique utilizing cation exchange resins was developed with a focus on concentrating low NH_4_^+^ samples for δ^15^N-NH_4_^+^ analysis in ice cores by Lamothe et al. (2023).^16^ Here, we have expanded upon this solid phase extraction methodology with a focus on concentrating laboratory standards and an in-house quality control (QC) solution as well as evaluated the method’s ability to extract NH_4_^+^ from marine aerosol samples that exhibit matrixes that impede the chemical conversion of NH_4_^+^ to N_2_O when using the well-established chemical method for δ^15^N-NH_4_^+^ analysis created by Zhang et al. (2007).^11^ These matrix interference issues were not addressed in Lamothe et al. (2023) study. We focused on optimizing the various parameters of the solid phase extraction technique (resin amount and extraction concentration) to achieve the highest possible precision and accuracy of δ^15^N for atmospheric samples. This work may also be a crucial step in developing robust methodologies for the accurate and precise δ^15^N characterization of NH_4_^+^ from other types of environments.

## Experimental Section

### Protocol Development

We targeted a 10 μM solution of NH_4_^+^ once samples were extracted from the resin columns because previous work has suggested that at this concentration, we can acquire precise δ^15^N measurements.^5,11,12^ Additionally, we targeted 10 mL of final eluent solution such that we aim to bind 100 nmol of NH_4_^+^. Previous work has suggested that Bio-Rad AG-50W can be used for concentrating dilute NH_4_^+^ samples using 0.6 g of resin and 5 mL of 1 M sodium chloride (NaCl) extraction solution.^16^ Here, we evaluated the amount of resin and the amount/concentration of NaCl for the complete capture and removal of NH_4_^+^ on the resin. Our evaluation criteria were (1) consistent production of generated N_2_O from samples and reference materials following the chemical conversion of NH_4_^+^ to N_2_O, (2) high precision/low standard deviations of the δ^15^N measurements of isotope reference materials and in house quality control sample (QC), and (3) a δ^15^N-NH_4_^+^ calibration slope of the isotope reference materials close to 2.0, as expected for conversion of NO_2_^–^ with sodium azide to N_2_O.^11,17^ The slope is calculated between the observed average NH_4_^+^ blank of the international standards to their δ^15^N reference values.

Note that in the initial protocol development phase, concentrations of NH_4_^+^ and NO_2_^–^ were determined using a discrete UV–Vis analyzer (colorimetrically via a Westco Smartchem 200; U.S. EPA Compliant Methods 350.1 and 353.2, respectively (Revision 2.0)).^18,19^ Reproducibility calculated from replicate measurements of quality control standards are typically ±2% and ±1% for NH_4_^+^ and NO_2_^–^, respectively, while limits of detection are approximately 0.3 and 0.2 μM for NH_4_^+^ and NO_2_^–^, respectively.^13^ With the established protocol, oxidized sample concentrations were checked via the expected peak areas of N_2_O on the isotope ratio mass spectrometer (IRMS).

### Cation Exchange Resin Preparation

**Figure 1:**
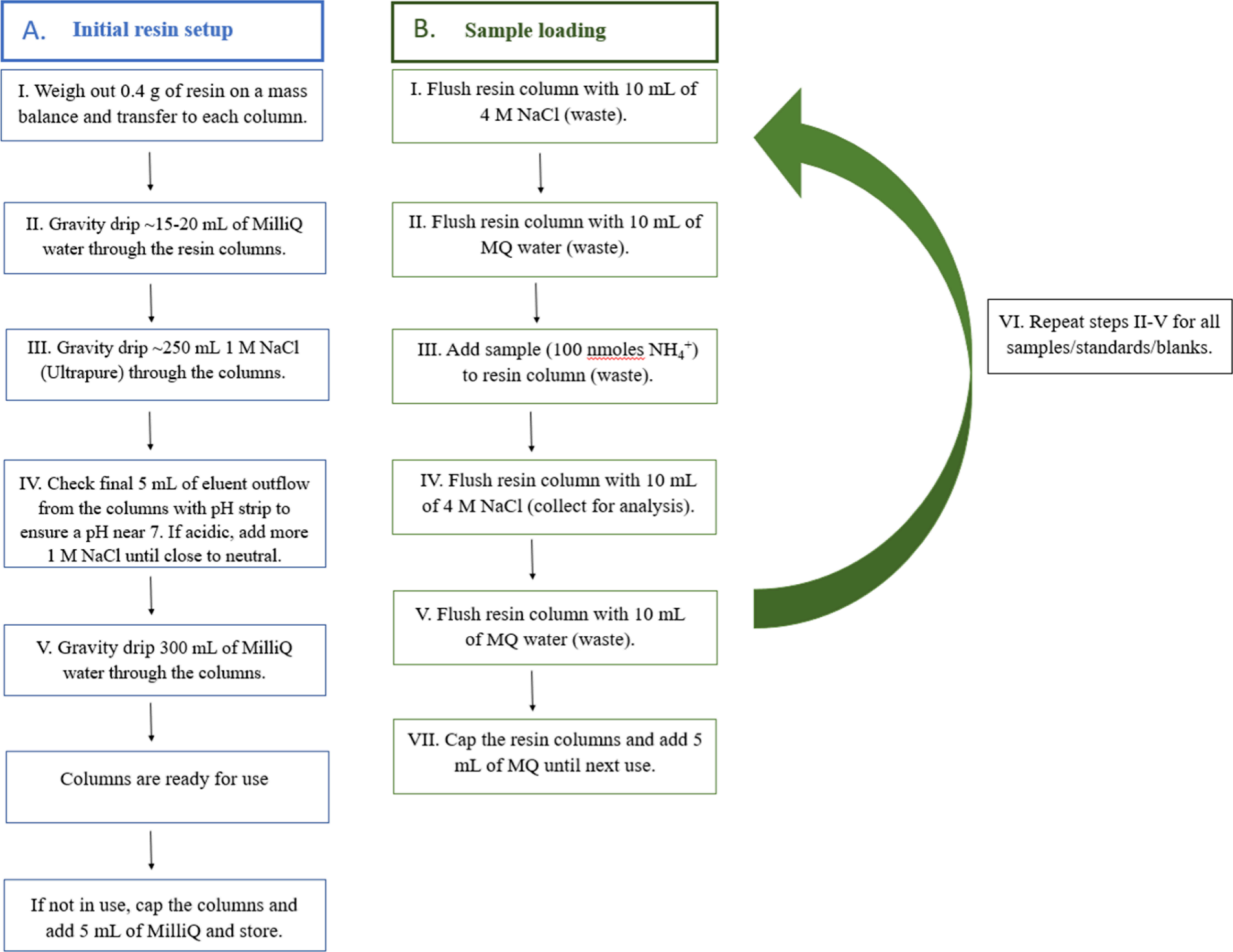
Flowchart of steps for (A) the initial resin column setup and (B) use of cation exchange columns to concentrate or purify NH_4_^+^ samples.

Bio-Rad AG-50W cation exchange resins (AG-50W-X2 Cation Exchange Resin, analytical grade, 50–100 mesh, hydrogen form, 0.6 Meq/mL) were utilized for concentrating NH_4_^+^ (aq). Resin columns were prepared by transferring the targeted resin mass to 10 mL Bio-Rad poly prep columns, which were attached to 250 mL funnels (Econo-Column Funnels). The control tests consisted of both 0.8 and 0.4 g resins tested with different NaCl concentrations such as 0.5, 1, 2, 3, and 4 M. (Prior to the publication of the Lamothe et al. (2023)^16^ study, we initially chose 0.8 g due to the 0.8 g/mL density of the resin; further testing (see below) revealed more accurate and consistent isotope values with 0.4 g (see below).) The resin was hydrated by gravity dripping ∼15–20 mL of ultraclean Milli-Q water (>18 MΩ) through the resin columns. The resin is initially in the hydron (H^+^) form and is converted to sodium (Na^+^) by gravity dripping ∼250 mL of 1 M NaCl (Thermo Scientific Sodium Chloride, ACS, Ultrapure) through the columns. The Bio-Rad AG 50W cation exchange resin has a higher relative selectiveness to NH_4_^+^ (1.95) compared to Na^+^ (1.5), such that the targeted NH_4_^+^ analyte will be effectively collected on the resin. The final 5–10 mL of eluent outflow from the columns is checked with pH strips to ensure a pH near 7, indicating that Na^+^ has replaced all H^+^ ions in the resin. If the pH remains acidic (pH < 5), an additional 50 mL of 1 M NaCl is passed through the resins. Once the pH of the outflow is close to neutral, 300 mL of Milli-Q water is then passed through the columns to thoroughly rinse them and expel excess Na^+^ (aq). Following these steps, the columns are then ready for use (Figure 1A). When not in use, the columns are stored in 5 mL of Milli-Q water, and the ends of the columns are capped (Poly-Prep Column Stack Cap and End Caps, Micro Bio-Spin chromatography columns) to keep the resin beds hydrated.

### Concentration Protocol

Once the resin columns were prepared and conditioned in the Na^+^ form, they were then utilized for the concentration of NH_4_^+^ (aq) samples in an alternating sample load and extraction/regeneration phase (Figure 1B). Prior to use, the stored resin columns were uncapped, and the Milli-Q used to keep the resins hydrated was removed by gravity dripping. Next, 10 mL of targeted NaCl concentrations were dripped through the columns followed by 10 mL of Milli-Q water, which was both collected and disposed of as waste. For the control tests, we targeted 0.5, 1, 2, 3, and 4 M NaCl, where we found 4.0 M NaCl to be the optimal concentration and utilized this concentration for the sample pretreatment of environmental samples.

The resins were then placed in the sample load phase (Figure 1B). Briefly, samples were allowed to gravity drip through the resin columns, and the outflow was collected and disposed of as waste. During the dripping process, the column funnels were covered with aluminum foil to limit contamination. After the samples were passed through the resins, the resins moved into the sample extraction/regeneration phase (Figure 1B). Briefly, 10 mL of 4 M NaCl was passed through the resin, which extracted the sample NH_4_^+^ initial bound to the resin and in the process regenerated the resin in the Na^+^ form. The NaCl eluent outflow containing the sample NH_4_^+^ was collected for subsequent δ^15^N analysis. After the NaCl extraction solution finished dripping, 10 mL of Milli-Q water was gravity-dripped through the columns to remove excess NaCl. The resins were then ready for the next sample loading phase. The sample loading and extraction/regeneration phases were repeated, until all samples for a particular batch of analyses were completed. In line with the identical treatment principle, for each batch analysis, NH_4_^+^ isotope reference materials (IAEA-N2 and USGS-25), a laboratory NH_4_^+^ quality-control (QC), and Milli-Q blanks were passed through the resin columns and processed the same way. The reference values for IAEA-N2 and USGS-25 were then used to calibrate the final δ^15^N values of the samples. Typically, the isotope reference materials, QC, and blanks were processed in quintet for each batch analysis. The standard deviations of repeated measures of the reference materials or samples are used as an indicator of precision, whereas the average QC values are used as an indicator for accuracy (i.e., the QC is treated as a sample, and its δ^15^N values are compared within and across batch analyses). Once all samples, standards, and blanks were processed through the resins, the columns were capped and hydrated, and stored (room temperature environment or refrigerator is acceptable) for subsequent use.

### Control Tests

We first evaluated the various parameters of the solid phase extraction method to ensure accurate δ^15^N-NH_4_^+^ values for the NH_4_^+^ isotope reference materials and laboratory QC at an initial [NH_4_^+^ (aq)] of 10 μM. Different combinations of the NaCl eluent concentrations (0.5, 1, 2, 3, and 4 M) and resin amounts (0.4 and 0.8 g) were tested. Once the optimal combination of resin amount and eluent concentration was determined, we evaluated the ability of the method to concentrate NH_4_^+^ (aq) for accurate and precise δ^15^N-NH_4_^+^ of the isotope reference materials and laboratory QC with initial [NH_4_^+^ (aq)] concentrations of 7.5, 5.0, 2.5, and 1.0 μM. For each concentration, 100 nmol of NH_4_^+^(aq) was captured such that the volume of the standards and QC that passed through the resin ranged from 10 to 200 mL. Additionally, blanks were determined using Milli-Q water passed through the resin at the same volume as the standard and QC sets. The NH_4_^+^ standards and QC were also analyzed for δ^15^N-NH_4_^+^ at 7.5, 5.0, 2.5, and 1.0 μM without the use of the pretreatment step (i.e., the cation exchange resin) to compare and further investigate the effectiveness of this resin method.

### Environmental Samples

Once the parameters of the solid-phase extraction protocol (resin bead volume and NaCl eluent concentration) were optimized, the method was applied to environmental samples. The method was initially utilized to compare with previously reported δ^15^N-NH_4_^+^ from aerosol samples collected from Changdao Island, China.^7^ These samples were previously analyzed for δ^15^N-NH_4_^+^ without the use of the resin concentration method because these samples exhibited no matrix interference upon NH_4_^+^ oxidation and had sufficiently elevated [NH_4_^+^ (aq)] (i.e., typically greater than 10 μM).^7^ These samples were analyzed in three different ways: (1) no pretreatment such that samples were thawed and represented their initial matrix (Milli-Q water extracts from aerosol filters), (2) no resin treatment but with samples made up in a 4 M NaCl matrix (e.g., samples diluted with a 4 M NaCl solution instead of pure Milli-Q water), and (3) complete processing of samples using the described pretreatment resin method. These tests were conducted to separately evaluate the impact of the 4 M NaCl matrix as well as potential resin effects on the δ^15^N analysis of aerosol samples.

Aerosol samples collected in Oahu, Hawaii and extracted in Milli-Q water were also analyzed for δ^15^N-NH_4_^+^ using this exchange resin methodology. Without the use of the sample pretreatment resin method, these samples failed to quantitatively oxidize to NO_2_^–^ (e.g., all samples were <60% oxidized) using the standard alkaline hypobromite solution, a critical first step for δ^15^N-NH_4_^+^ analysis using a coupled chemical oxidation and reduction methodology that converts NH_4_^+^ to N_2_O^11^. The poor oxidation yields were confirmed from the measurement of NO_2_^–^ after the NH_4_^+^ oxidation step using a discrete UV–Vis analyzer. The ability of the resin concentration method to remove the apparent matrix effect limiting NH_4_^+^ oxidation was evaluated from analyzing the N_2_O peak areas on a continuous flow IRMS. Since we are injecting the same amount of material (i.e, 10 nmol of NO_2_^–^) for samples and standards, we would expect consistent N_2_O peak areas using the IRMS (around 20 Vs in our case but could differ between laboratories depending on the sensitivity of the IRMS instrument).

### Determination of δ^15^N-NH_4_^+^

All NH_4_^+^ samples, standards, QCs, and laboratory blanks were oxidized to NO_2_^–^ using a chemical method involving hypobromite (BrO^–^) in an alkaline solution, which was synthesized as previously described.^11^ It is noted that the BrO^–^ solution typically experiences minimal interference from dissolved organic nitrogen (DON).^11^ Though we do note that the role of DON as a potential interference in the δ^15^N analysis of NH_4_^+^ could be dependent on the environment in which the samples are collected. This requires attention due to the potential for DON oxidation to NO_2_^–^, similar as the intended target of NH_4_^+^ oxidation. Thus, we recommend careful evaluation of the product NO_2_^–^ and/or N_2_O to evaluate the role of DON oxidation as a potential interference. If greater than expected NO_2_^–^ or N_2_O is observed, this could potentially implicate the role of DON. For the considered aerosol samples, we never encountered more than expected NO_2_^–^ and/or N_2_O and thus assume the presence of DON is negligible for these types of samples. This chemical method was shown to be more accurate for δ^15^N-NH_4_^+^ analysis compared to the microdiffusion method.^20^ Briefly, samples and standards were transferred into 50 mL vials that are acid washed, rinsed with Milli-Q water, and baked at 500 °C for 4 h prior to use. Next, 2 mL of the BrO^–^ oxidizing reagent was added to each of the sample and standard 50 mL vials, capped, and allowed to react overnight. Then 0.5 mL of 1 M sodium arsenite is added to each vial to remove the remaining BrO^–^. After oxidation, 10 nmol of the generated NO_2_^–^ were then transferred into 20 mL vials, crimp-capped with PTFE/butyl septa, and flushed with helium gas for a minimum of 10 min. The NO_2_^–^ was then reduced to N_2_O through the addition of 2 mL of 2 M sodium azide buffered in 40% acetic acid solution.^11^ After a minimum of 0.5 h, the samples were neutralized by using 6 M sodium hydroxide. Samples were then analyzed for the δ^15^N–N_2_O composition via an automated N_2_O extraction system coupled with a continuous flow IRMS for determination at *m*/*z* 44, 45, and 46. Two internationally recognized δ^15^N-NH_4_^+^ reference materials (composed of ammonium sulfate), IAEA-N2 (δ^15^N = 20.4 ± 0.2‰) and USGS-25 (δ^15^N = −30.3 ± 0.3‰)^21^^,^^22^ and an internal QC (δ^15^N = −1.0 ± 0.5‰) were analyzed during every run. The final δ^15^N-NH_4_^+^ is determined by correction for isobaric influences, blank contribution, and calibrating the unknown samples and QC to the internationally recognized standards, as previously described.^12,13^ The authors note that no unexpected safety hazards were encountered with this method.

## Results and Discussion

### Optimizing NaCl Extraction Concentration

**Figure 2:**
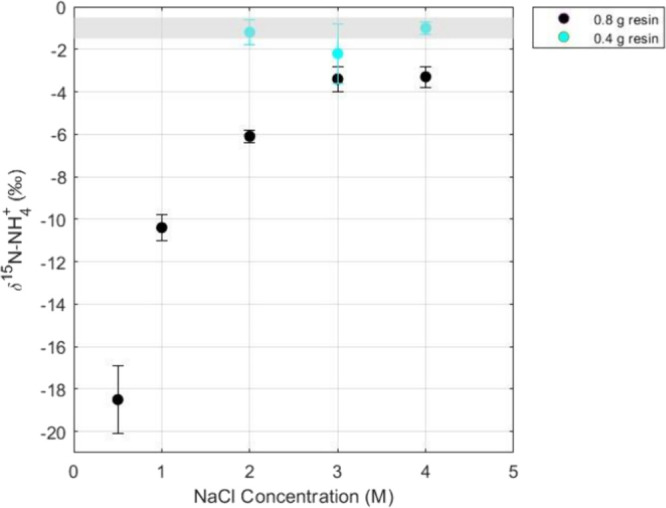
Average QC δ^15^N-NH_4_^+^ values post-cation exchange resin with varying concentrations of NaCl extraction solution (*n* = 10). Black circles are the average δ^15^N-NH_4_^+^ using 0.8 g resin, and blue circles are the average δ^15^N-NH_4_^+^ using 0.4 g resin. Shaded region indicates the expected QC δ^15^N-NH_4_^+^ range. Error bars represent ±1σ.

For all tested resin amounts and NaCl extraction concentrations, besides 0.5 M NaCl, the standard deviations of the NH_4_^+^ standard solutions (i.e., IAEA-N2 and USGS-25) were generally within 1‰ (Table 1). However, we found that the accuracy of the calibrated δ^15^N-NH_4_^+^ of QC was strongly dependent on the solid-phase extraction parameters (Figure 2). Only the combinations that utilized 0.4 g resin resulted in a δ^15^N-NH_4_^+^ of the QC that overlapped with its expected value, with two out of three of those combinations producing an average that fits the expected range (2 and 4 M NaCl; Figure 2).

**Table 1:** Average Standard (IAEA-N2 and USGS-25) and QC δ^15^N-NH_4_^+^ Values Post-Cation Exchange Resin with Varying Concentrations of NaCl Extraction Solution and Resin Amount (*x̅* ± 1σ (*n*))^a^

**NaCl (M)**	**resin (g)**	**δ**^**15**^**N (IAEA-N2) (**‰)	**δ**^**15**^**N (USGS-25) (**‰)	**δ**^**15**^**N (QC) (**‰)	**standards& QC peak area (Vs)**	**blank peak area (Vs)**	**slope**^b^
0.5	0.8	20.4 ± 3.3 (8)	–30.3 ± 2.5 (10)	–18.5 ± 1.6 (10)	15.0 ± 1.7	3.2 ± 0.2 (5)	2.7 ± 0.07
1	0.8	20.4 ± 0.7 (10)	–30.3 ± 1.1 (10)	–10.4 ± 0.6 (10)	15.3 ± 1.3	2.4 ± 0.3 (5)	2.4 ± 0.02
2	0.8	20.4 ± 0.5 (10)	–30.3 ± 0.9 (10)	–6.0 ± 0.3 (10)	16.1 ± 0.8	3.6 ± 0.3 (5)	2.1 ± 0.01
3	0.8	20.4 ± 0.5 (10)	–30.3 ± 0.8 (10)	–3.4 ± 0.6 (10)	14.7 ± 0.8	2.7 ± 0.3 (5)	2.2 ± 0.01
4	0.8	20.4 ± 0.7 (10)	–30.3 ± 0.7 (10)	–3.3 ± 0.6 (8)	14.5 ± 1.6	2.9 ± 0.4 (4)	2.0 ± 0.01
2	0.4	20.4 ± 0.6 (10)	–30.3 ± 0.7 (10)	–1.2 ± 0.6 (10)	16.3 ± 1.3	2.8 ± 0.1 (5)	2.1 ± 0.01
3	0.4	20.4 ± 1.0 (10)	–30.3 ± 1.4 (10)	–2.2 ± 1.4 (10)	14.3 ± 1.0	2.4 ± 0.2 (5)	2.1 ± 0.02
4	0.4	20.4 ± 1.0 (9)	–30.3 ± 0.6 (9)	–1.0 ± 0.3 (10)	16.3 ± 1.8	3.5 ± 1.0 (5)	2.0 ± 0.02

a Combined average standard & QC peak areas (Vs), blank peak averages (Vs), and calculated corrected slopes are included. All standards and QCs were targeted at 10 μM NH_4_^+^.

b A slope of 2.0 is expected due to the conversion of NO_2_^–^ with sodium azide to N_2_O.

The δ^15^N-NH_4_^+^ of the QC for the 3 M NaCl set was the most variable (1σ = ± 1.4‰;*n* = 10), compared with the 2 M NaCl (1σ = ± 0.6‰;*n* = 10) and 4 M NaCl (1σ = ± 0.3‰;*n* = 9) sets. While the 2 M NaCl extraction solution produced an accurate and reasonably precise δ^15^N-NH_4_^+^ of the QC, we recommend utilizing 0.4 g of resin and 4 M NaCl as the optimal extraction solution concentration for the NH_4_^+^ solid phase extraction methodology as these parameters resulted in the highest accuracy and best precision for δ^15^N-NH_4_^+^. Additionally, the use of 4 M NaCl will provide a buffer (e.g., excess Na^+^ without impeding subsequent oxidation).

Not only are these optimal parameters different from Lamothe et al. (2023),^16^ who utilized 0.6 g of resin and 5 mL of 1 M NaCl, but the concentration of NaCl required in this study is higher than expected based off the Biorad cation exchange resin instruction manual.^23^ The manual suggests that changing the resin from NH_4_^+^ (relative selectivity of 1.95) to Na^+^ (relative selectivity of 1.5) should require no more than two bed volume of 1 M NaCl and converting the resin volume from Na^+^ to NH_4_^+^ should require two to three bed volumes. However, this study indicated that 1 M NaCl is too low of a concentration to successfully extract the NH_4_^+^ from the resin for accurate δ^15^N-NH_4_^+^ measurement, which instead required 10 mL (∼20 bed volumes) of 4 M NaCl to obtain accurate isotopic results (Table 1, Figure 2).

### Concentrating NH_4_^+^

**Figure 3:**
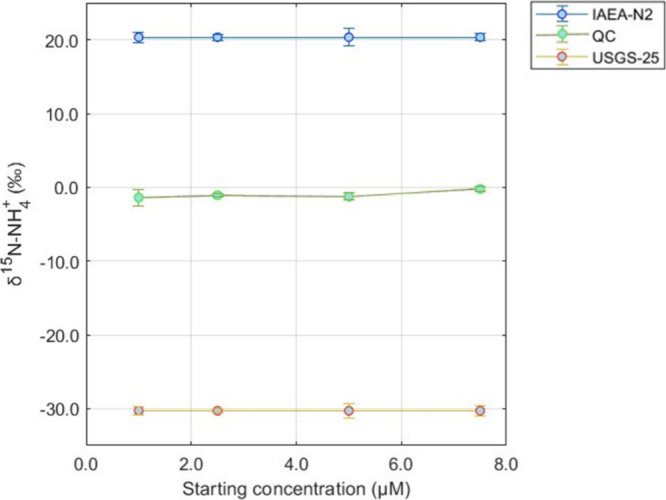
Standard (IAEA-N2 and USGS-25) and QC δ^15^N-NH_4_^+^ averages at different concentrations were concentrated to 10 μM through a cation exchange resin (0.4 g) and extracted with 10 mL of 4 M NaCl. Error bars represent ±1σ. Data are from Table 2.

Utilizing the optimized resin volume and extraction parameters, the solid-phase extraction method was evaluated for its ability to concentrate NH_4_^+^ samples for δ^15^N-NH_4_^+^ analysis. Overall, the standard deviations of δ^15^N-NH_4_^+^ values for the isotope reference materials and QC were below 1.3‰, with most being below 1.0‰ (Table 2; Figure 3). Additionally, there were consistent IRMS responses (e.g., δ^15^N-NH_4_^+^ peak areas) for the various concentration targets (Table 2), indicating a complete oxidation of NH_4_^+^ to N_2_O. The calibration slope between the observed NH_4_^+^ values and the expected reference values was expected to be near ∼2.0 (as one N atom in the product of N_2_O is from the azide reagent),^17^ which was observed for each [NH_4_^+^] set (Table 2). This criterion was used to evaluate the performance of solid phase extraction method conditions along with low standard deviations of the isotope reference materials. Further, outliers in the data set were tested using an outlier test for peak areas and samples were removed if peak areas were significantly high or low (±2σ). For all sets, average peak areas of blanks were <22% of average sample and standard peak areas. Although a blank was present, it was consistent, reproducible, and correctable.

**Table 2:** Average Corrected Standard (IAEA-N2 and USGS-25) and QC δ^15^N-NH_4_^+^ Values Post-Cation Exchange Resin (0.4 g) Extracted with 10 mL of 4 M NaCl All Concentrated to 10 μM (*x̅* ± 1σ (*n*))^a^

**NH**_**4**_^**+**^**(μM)**	**δ**^**15**^**N (IAEA-N2) (**‰)	**δ**^**15**^**N (USGS-25) (**‰)	**δ**^**15**^**N (QC) (**‰)	**standard& QC peak area (Vs)**	**blank peak area (Vs)**	**slope**
7.5	20.4 ± 0.5 (10)	–30.3 ± 0.7 (10)	–0.2 ± 0.4 (10)	17.9 ± 1.5	2.7 ± 0.3 (5)	2.2 ± 0.01
5.0	20.4 ± 1.2 (10)	–30.3 ± 1.0 (10)	–1.3 ± 0.5 (9)	19.1 ± 1.8	3.5 ± 0.3 (5)	2.0 ± 0.02
2.5	20.4 ± 0.4 (9)	–30.3 ± 0.3 (10)	–1.1 ± 0.2 (10)	17.5 ± 2.1	2.6 ± 0.03 (5)	2.1 ± 0.01
1.0	20.4 ± 0.7 (10)	–30.3 ± 0.6 (10)	–1.4 ± 1.1 (10)	21.3 ± 3.2	2.9 ± 1.5 (4)	2.5 ± 0.02

a Combined average standard & QC peak areas (Vs), blank peak averages (Vs), and calculated corrected slopes are included.

The blank did not increase with increasing NaCl concentration, indicating that this blank is not derived from the NaCl extraction solution (as either NH_4_^+^ or NO_2_^–^). As Milli-Q water injections (without the alkaline hypobromite oxidation reagent) were below detection limits, it was determined that the sodium/azide/acetic acid reagent also did not contribute significantly to the blank. Further, the reagent blank did not increase for lower initial NH_4_^+^ concentrations that required an increased volume of Milli-Q water passing through the resin, which indicated a lack of significant NH_4_^+^ blank in the laboratory Milli-Q water. Therefore, the most likely source of the blank is the alkaline hypobromite reagent.

### Low [NH_4_^+^] Samples without Resins

**Figure 4:**
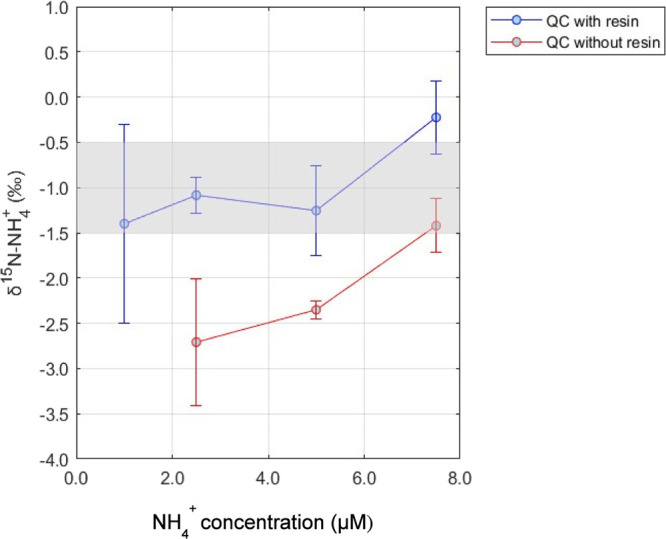
QC δ^15^N-NH_4_^+^ averages at different concentrations that were concentrated to 10 μM through a cation exchange resin (0.4 g) and extracted with 10 mL of 4 M NaCl (blue line) compared to QC δ^15^N-NH_4_^+^ averages at different concentrations that did not get concentrated and did not use the pretreatment resin step (red line). Shaded region indicates expected QC δ^15^N-NH_4_^+^ range. Error bars represent ±1σ. Data are from Tables 2 and 3

The δ^15^N measurements for NH_4_^+^ concentrations below 10 μM without the use of the cation resin sample pretreatment revealed QC δ^15^N-NH_4_^+^ outside of the expected range (−1.0 ± 0.5‰) for 2.5 and 5.0 μM and within the expected range for 7.5 μM (Table 3, Figure 4).

**Table 3:** Average Corrected Standard (IAEA-N2 and USGS-25) and QC δ^15^N-NH_4_^+^ Values at Various [NH_4_^+^] without Use of the Pre-treatment Resin Step (*x̅* ± 1σ (*n*))^a^

**NH**_**4**_^**+**^**(μM)**	**δ**^**15**^**N (IAEA-N2) (**‰)	**δ**^**15**^**N (USGS-25) (**‰)	**δ**^**15**^**N (QC) (**‰)	**standard& QC peak area (Vs)**	**blank peak area (Vs)**	**slope**
7.5	20.4 ± 1.1 (3)	–30.3 ± 0.6 (5)	–1.4 ± 0.3 (5)	22.8 ± 1.5	3.7 ± 0.4 (5)	2.0 ± 0.03
5.0	20.4 ± 0.5 (5)	–30.3 ± 1.5 (4)	–2.4 ± 0.1 (3)	21.7 ± 1.5	4.7 ± 0.5 (5)	2.0 ± 0.03
2.5	20.4 ± 0.5 (5)	–30.3 ± 3.4 (4)	–2.7 ± 0.7 (3)	18.0 ± 2.7	2.9 ± 0.5 (5)	2.1 ± 0.07
1.0	N/A (5)	N/A (5)	N/A (5)	N/A	3.6 ± 0.8 (5)	0.0 ± 0.1

a Combined average standard & QC peak areas (Vs), blank peak averages (Vs), and calculated corrected slopes are included.

As the concentration decreases, the uncertainty in calibration increases. This is most likely what drove the QC to have a different calibrated value compared to its expected value (Table 3).

It is noted that for the 1.0 μM set, failure to oxidize was consistent across the run (Table 3). This 1.0 μM set was replicated a second time with similar results leading to oxidation failures (average combined standard peak area of 4.4 ± 1.8 (*n* = 25) and average blank peak area of 2.4 ± 0.8 (*n* = 4)). Therefore, opting not to use the cation exchange resins for samples below 7.5 μM can lead to potential δ^15^N biases and in some cases (i.e., concentration below 1 μM) an inability to measure δ^15^N (Table 3, Figure 4). The cation exchange resin sample pretreatment enabled accurate concentration of 1.0, 2.5, 5.0, and 7.5 μM NH_4_^+^ standards and QCs for precise and accurate δ^15^N determination and is recommended to be applied for NH_4_^+^ samples with concentrations below 10 μM (Figures 3 and 4).

### Application to Marine Ammonium Aerosol Samples

Here, we evaluated the resin methods ability to accurately reproduce previously reported δ^15^N-NH_4_^+^ in marine aerosol samples from Changdao Island, China.^7^ These samples, during and after the original analyses, were stored in a freezer to preserve chemical composition. When analyzing the four previously measured aerosol samples, all three methods were shown to determine δ^15^N-NH_4_^+^ values with excellent comparison to their expected δ^15^N-NH_4_^+^ values (Table 4). Note that the measured NH_4_^+^ concentrations were >100 μM for each sample. The excellent reproducibility of δ^15^N-NH_4_^+^ from the samples previously reported as sample rerun after storage in a freezer for approximately 3.5 years indicates cryostorage is suitable for preserving aerosol NH_4_^+^ samples for δ^15^N analysis.

**Table 4:** δ^15^N-NH_4_^+^ Values (± 1σ Where Applicable) of Four Previously Measured Aerosol Samples (Sample ID **China 1–4**) from Changdao Island, China along with Corrected QC δ^15^N-NH_4_^+^ Values (± 1σ) All Diluted to 10 μM NH_4_^+^^a^

**sample ID**	**previously reported δ**^**15**^**N (**‰**)**	***n***	**remeasured δ**^**15**^**N (**‰)	***n***	**δ**^**15**^**N (NaCl matrix) (**‰**)**	***n***	**δ**^**15**^**N (pretreatment resin step) (**‰**)**	***n***
**China 1**	2.5 ± 0.0	2	3.3	1	3.7	1	3.7	1
**China 2**	–8.8 ± 0.3	2	–8.6	1	–7.6 ± 0.1	2	–9.1	1
**China 3**	14.6 ± 0.0	2	14.4 ± 0.2	2	14.2 ± 0.6	2	14.0 ± 0.2	2
**China 4**	15.4 ± 0.3	2	15.4 ± 0.1	2	15.8 ± 0.03	2	15.0 ± 0.1	2
**QC**	–1.2 ± 0.3	12	–1.1 ± 0.3	3	–0.6 ± 0.2	3	–1.2 ± 0.2	3

a These samples and QC were run three separate ways: the same method as the previously reported isotope values (Milli-Q matrix and no resin), with a 4 M NaCl matrix and no resin, and with a Milli-Q matrix and extracted from the resin columns with 10 mL of 4 M NaCl.

**Figure 5:**
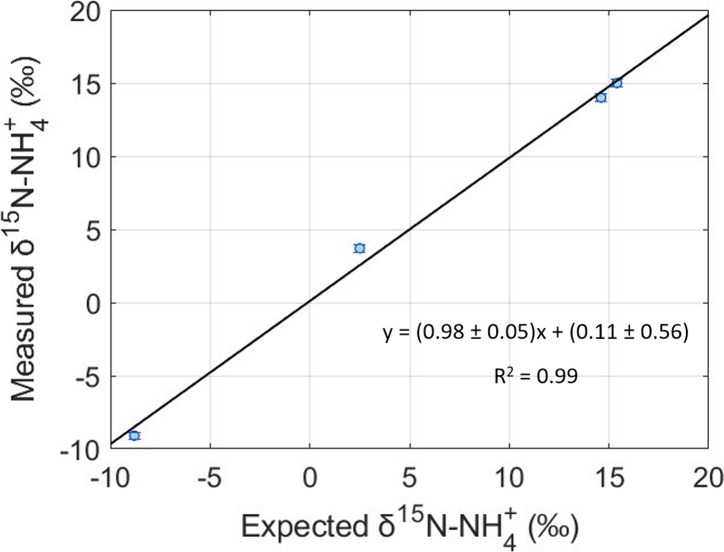
Expected vs measured δ^15^N-NH_4_^+^ values of four aerosol samples collected on Changdao Island, China. Measured values represent treated samples post resin and extracted with 10 mL of 4 M NaCl. Error bars represent ±1σ.

China samples 1, 3, and 4 are duplicates (analyzed twice in the same run), and sample 2 is a replicate (analyzed twice over two separate runs). These four samples were run over three separate runs in total and QCs from all three runs were averaged (*n* = 12; Table 4; “previously reported δ^15^N (‰)” column). For the treated samples that utilized the resin methodology, the pooled standard deviation for sample duplicates is <0.3‰. Additionally, average peak areas of blanks (*n* = 3) were consistently low (<17% of average sample and standard peak areas). Overall, the expected values versus the measured values were very strongly linear (Figure 5), and the average differences between the expected (previously reported) δ^15^N-NH_4_^+^ and the measured δ^15^N-NH_4_^+^ with the pretreatment resin step is ≤0.6‰ (*n* = 4 pairs). This comparison indicates that treatment via the resin method and the extraction matrix does not induce significant δ^15^N biases to the environmental samples.

### Utilizing the Resin Method to Separate NH_4_^+^ from Samples with Matrix Interferences

**Figure 6:**
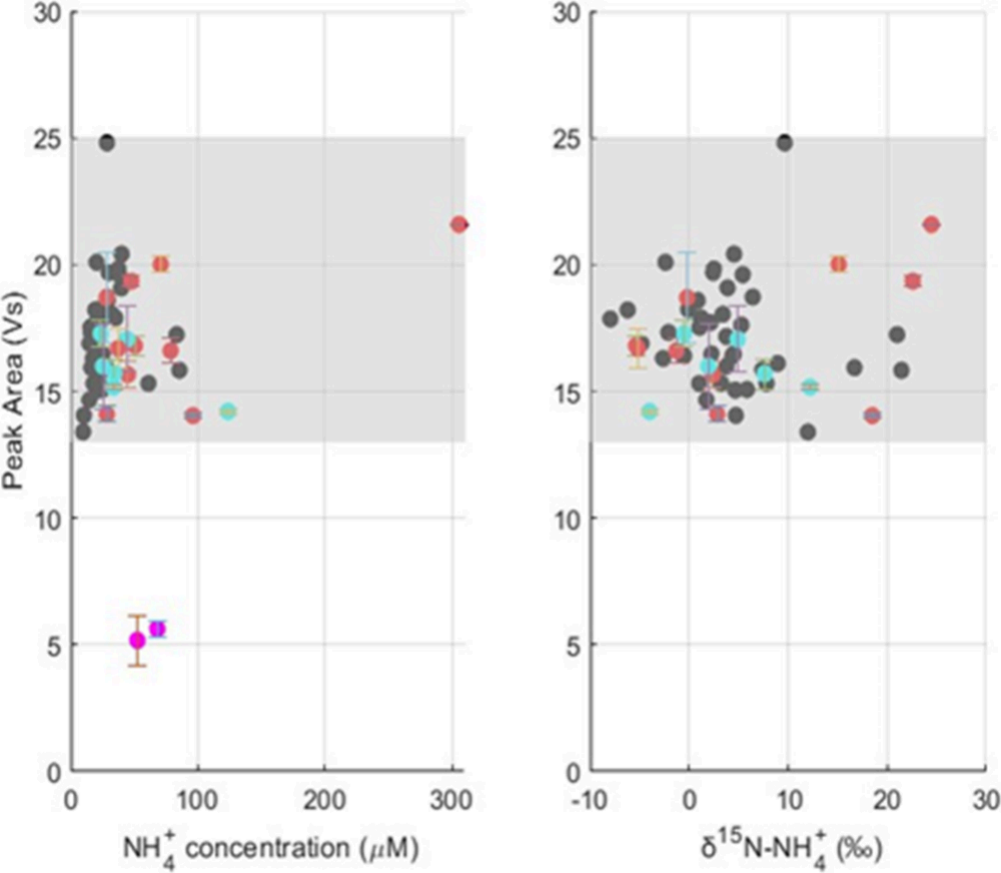
(A) NH_4_^+^ concentration (μM) of Hawaii marine aerosol samples versus peak area (Vs) from the IRMS. (B) δ^15^N-NH_4_^+^ (‰) of Hawaii marine aerosol samples vs peak area (Vs) from the IRMS. For both (A) and (B), shaded regions depict the acceptable peak range for proper oxidation for these samples. The shaded region quantitatively representing successful oxidation, was determined by creating a range from taking the lowest peak area average minus the standard deviation and the highest peak area average plus the standard deviation from Tables 1–3. The legend refers to peak area analyses where black circles = single samples, blue circles = replicate samples, red circles = duplicate samples, and pink circles = samples run as a duplicate and replicate. Error bars represent ±1σ for peak area.

Previous marine NH_4_^+^ aerosol samples from Hawaii demonstrated significant difficulties in being oxidized to NO_2_^–^ without using the resin sample pretreatment methodology. These samples had sufficiently high NH_4_^+^ concentrations that ranged from 9 to 306 μM. These samples were processed with the hypobromite alkaline reagent where the NH_4_^+^ failed to completely oxidize to NO_2_^–^. For example, 14 samples that yielded lower than expected [NO_2_^–^] (<60% converted) were processed for their isotopic composition. In addition to revealing incomplete oxidation through lower than anticipated [NO_2_^–^], they were further shown to be only partially oxidized based on the low N_2_O IRMS peak areas where conversion ranged from 28 to 72% of that expected. Additionally, poor isotope accuracy and precision were found with the lower than expected conversion to NO_2_^–^, such that a direct comparison between the results with and without the resin yielded a slope of 0.63 and an *R*^2^ of 0.67 (*n* = 14). However, when processing these Hawaii samples (*n* = 57) using the solid phase extraction methodology, 96% successfully oxidized (Figure 6A).

Duplicates (*n* = 10 pairs) and replicates (*n* = 6 pairs) had a pooled standard deviation of 0.4 and 0.5‰, respectively, which is comparable to the standard deviation values of δ^15^N for the international standards (Table 5). Additionally, average peak areas of blanks (*n* = 3 per run) were consistently low enough to be adequately corrected for (<29% of average sample and standard peak areas). Furthermore, the standard/QC averages and low standard deviations in each run, along with the consistent sample peak areas (Table 5), provide confidence that the resin sample pretreatment method enabled the successful measurement of accurate and precise δ^15^N-NH_4_^+^ for the Hawaii aerosol samples with a difficult sample matrix that originally impeded oxidation (Figure 6B).

**Table 5:** Average Corrected Standard and QC δ^15^N-NH_4_^+^ Values Post-Cation Exchange Resin (0.4 g) Extracted with 10 mL of 4 M NaCl for Unknown Hawaii Marine Aerosol Sample Runs (*x̅* ± 1σ (*n*))^a^

**run**	**δ**^**15**^**N (IAEA-N2) (**‰)	**δ**^**15**^**N (USGS-25) (**‰)	**δ**^**15**^**N (QC) (**‰)	**standard& QC peak area (Vs)**	**blank peak area (Vs)**	**slope**
1	20.4 ± 0.3 (2)	–30.3 ± 0.4 (3)	–1.4 ± 0.6 (3)	19.2 ± 0.9	5.0 ± 0.9 (3)	1.9 ± 0.02
2	20.4 ± 0.7 (6)	–30.4 ± 0.5 (6)	–1.2 ± 0.3 (6)	22.0 ± 1.1	5.3 ± 0.1 (3)	2.0 ± 0.02
3	20.4 ± 1.1 (4)	–30.3 ± 0.6 (4)	–0.7 ± 0.2 (4)	21.0 ± 1.2	3.7 ± 0.1 (3)	2.1 ± 0.03
4	20.4 ± 0.7 (4)	–30.3 ± 0.8 (4)	–2.0 ± 0.5 (4)	21.0 ± 1.9	4.3 ± 0.2 (3)	2.1 ± 0.03

a Combined average standard & QC peak areas (Vs), blank peak averages (Vs), and calculated corrected slopes are included.

Identifying the exact role of the cation exchange in separating out potential matrix interferences is beyond the scope of this work. While it is difficult to know what interferences impact NH_4_^+^ oxidation and how treating samples through the cation exchange resin removes these interferences, we speculate that this may be related to the presence of organics. The elevated concentration of the eluent extraction (4 M NaCl), which does not appear to impact oxidation, implies that inorganic ions do not have a significant impact on NH_4_^+^ oxidation. The cation exchange resin should only bind the sample cations, and it would be expected that most of the organic material (barring organic cations such as amines) within the sample should pass through the resin without being bound and subsequently extracted. We note that two samples still did not yield the expected N_2_O peak areas (Figure 6A). These samples were reprocessed through the resin methodology using a higher sample volume to potentially account for an incorrect starting NH_4_^+^ concentration, but this still yielded lower than expected N_2_O peak areas. It is unclear why these samples were still unable to be run successfully for δ^15^N-NH_4_^+^, though potentially a separate interference that the cation exchange resins were not able to remove is responsible, such as elevated levels of organic cations. Still, the resin sample pretreatment method significantly expands the ability to utilize the hypobromite/sodium azide chemical method for δ^15^N-NH_4_^+^ analysis of low concentration samples or samples with matrix interferences.

## Conclusions

The δ^15^N-NH_4_^+^ of environmental samples can be difficult to analyze due to low concentrations and potential matrix interferences that prevent necessary oxidation of NH_4_^+^ to N_2_O using widely employed chemical oxidation methods (e.g., a BrO^–^ oxidizing reagant). To combat some of these issues, we present a purification methodology for robust δ^15^N analyses of atmospheric NH_4_^+^ samples. This is achieved via a sample pretreatment step utilizing a solid phase extraction technique with cation exchange resins. In this method, 0.4 g of cation exchange resin (AG-50W) was used per column, and samples/standards/blanks were extracted with 10 mL of 4 M NaCl, parameters that were optimized through a series of control tests. These resins were able to successfully concentrate samples from 1.0 to 10 μM, which was tested on two international standards (IAEA-N2 and USGS-25) and an in-house quality control (QC) solution. Without use of this pretreatment step, the δ^15^N analyses of NH_4_^+^ samples below 7.5 μM were shown to be unreliable.

Additionally, samples from Changdao Island, China (*n* = 4), which were previously measured for δ^15^N-NH_4_^+^ and experienced no matrix interference issues, yielded excellent and comparable results with and without the use of the resins (*R*^2^ = 0.99). The average difference between the expected (previously reported) δ^15^N-NH_4_^+^ and the measured δ^15^N-NH_4_^+^ with the pretreatment resin step is ≤0.6‰. Importantly, this new methodology was successful on marine aerosol samples from Oahu, Hawaii (*n* = 57) that displayed matrix interference issues where the NH_4_^+^ could not previously be oxidized. Using this cation exchange resin methodology, 96% of samples successfully oxidized, and the pooled standard deviations for duplicate (*n* = 10) and replicate (*n* = 6) pairs were 0.4 and 0.5‰, respectively. Therefore, we recommend utilizing this sample pretreatment methodology for all environmental NH_4_^+^ samples to obtain accurate and precise δ^15^N measurement.

## Data Availability

The data from this study is available at https://doi.org/10.7910/DVN/ZQFLOJ.
